# *TLR9* 2848 GA Heterozygotic Status Possibly Predisposes Fetuses and Newborns to Congenital Infection with Human Cytomegalovirus

**DOI:** 10.1371/journal.pone.0122831

**Published:** 2015-04-06

**Authors:** Wioletta Wujcicka, Edyta Paradowska, Mirosława Studzińska, Zuzanna Gaj, Jan Wilczyński, Zbigniew Leśnikowski, Dorota Nowakowska

**Affiliations:** 1 Scientific Laboratory of Center of Medical Laboratory Diagnostics, Polish Mother’s Memorial Hospital—Research Institute, Lodz, Poland; 2 Department of Perinatology and Gynecology, Polish Mother's Memorial Hospital—Research Institute, Lodz, Poland; 3 Laboratory of Molecular Virology and Biological Chemistry, Institute of Medical Biology, Polish Academy of Sciences, Lodz, Poland; University of Regensburg, GERMANY

## Abstract

**Background:**

Some single nucleotide polymorphisms (SNP), located in *Toll-like receptor* (*TLR*) genes, were reported to be associated with human cytomegalovirus (HCMV) infections. The study was aimed to assess the correlation of SNPs at *TLR4* and *TLR9* genes with the occurrence of congenital cytomegaly, based on available samples.

**Methods:**

Reported case-control study included both HCMV infected and non-infected fetuses and newborns. The specimens were classified to the molecular analyses, based on serological features of the recent infection and HCMV DNAemia in body fluids. *TLR* SNPs were studied, using multiplex nested PCR-RFLP assay, and determined genotypes were confirmed by sequencing. Hardy-Weinberg equilibrium was assessed for the identified genotypes. The linkage disequilibrium was also estimated for *TLR4* SNPs. A relationship between the status of *TLR* genotypes and congenital cytomegaly development was estimated, using a logistic regression model.

**Results:**

Hardy Weinberg equilibrium was observed for almost all SNPs, both infected and non-infected patients, with exception of *TLR4* 896 A>G polymorphism in the control group (P≤0.050). *TLR4* 896 A>G and 1196 C>T SNPs were found in linkage disequilibrium in both study groups (P≤0.050). The CC genotype at *TLR4* 1196 SNP and the GA variant at *TLR9* 2848 G>A SNP were significantly associated with HCMV infection (P≤0.050). The risk of congenital cytomegaly was higher in heterozygotes at *TLR9* SNP than in the carriers of other genotypic variants at the reported locus (OR 4.81; P≤0.050). The GC haplotype at *TLR4* SNPs and GCA variants at *TLR4* and *TLR9* SNPs were significantly associated with HCMV infection (P≤0.0001). The ACA variants were more frequent among fetuses and neonates with symptomatic, rather than asymptomatic cytomegaly (P≤0.0001).

**Conclusions:**

*TLR4* and *TLR9* polymorphisms may contribute to the development of congenital infection with HCMV in fetuses and neonates. The *TLR9* 2848 GA heterozygotic status possibly predisposes to HCMV infection, increasing the risk of congenital cytomegaly development.

## Introduction

Human cytomegalovirus (HCMV) causes the most common intrauterine infections, affecting approximately 40% to 100% of pregnant women [1–5]. Vertical transmission of the virus from mother to fetus *via* the placenta occurs with the incidence rate of 30–40% in women primary infected with HCMV, while revealing 0.2–2.2% incidence rate in cases of recurrent infections [6–9]. Congenital infections of fetuses may be both asymptomatic and symptomatic with severe symptoms, including microcephaly, ventriculomegaly, increased periventricular echogenicity and calcifications [6, 10]. Clinical symptoms are observed in about 10% to 15% of congenitally infected neonates, out of whom, 85% to 90% demonstrate psychomotor and mental retardation [6, 11]. In addition, children with asymptomatic cytomegaly at birth (85% to 90%) may develop disease symptoms, including hearing impairment and difficulties in learning during the first months or, more often, in the first few years of life [6, 9, 12].

Regarding the non-specific immunity to HCMV infection, a crucial role was assigned to Toll-like receptors (TLRs) [13–16]. The expression of genes, encoding TLR2 and endosomal TLR3 and TLR9 molecules, as well as scavenger receptor A type 1 (SR-A1), tyrosine-protein kinase Lyn, IL-12 p35 subunit, TIR domain containing adaptor-inducing interferon-beta (TRIF), interferon regulatory factor 3 (IRF-3) and interferon beta (IFN-ß) were observed to be induced after infection of human acute monocytic leukemia cell line THP1 and foreskin fibroblast cell lines with HCMV [17, 18]. In foreskin fibroblasts and ectocervical tissue, ligands for TLR3 (poly I:C) and TLR4 (LPS) inhibited the HCMV infection, inducing the secretion of interleukin 8 (IL-8) and IFN-ß (^12^). TLR3 and TLR9 were also reported to activate the transcription of pro-inflammatory cytokines, although SR-A1 mediated the virus sensing process [18, 19]. TLR9 expression was strongly induced in the primary fibroblasts, infected with HCMV [14, 17].

Among the genetic markers, possibly associated with HCMV infections, the SNPs were reported, located in *TLR* genes. [20–22]. The CC genotype at SNP rs3804100 in the *TLR2* gene was correlated with congenital HCMV infection, although no association was observed with the course of cytomegaly [22]. In the same group of infected children, the AG genotype at SNP rs1898830 in the *TLR2* gene was more frequent than in the group of non-infected children. On the other side, no relationship was observed between SNPs in the *TLR4* and *TLR9* genes and congenital HCMV infection or cytomegaly [22]. However, other studies showed some role of SNPs in *TLR2*, *TLR3*, *TLR4*, *TLR7* and *TLR9* genes in HCMV infections [20, 21, 23, 24]. Renal transplant recipients (RTRs), who carried *TLR4* 896 A>G and *TLR4* 1196 C>T SNPs, demonstrated more frequent opportunistic infections and cytomegaly [17, 23]. Also *TLR9*–1237 SNP was marginally associated with recurrent urinary infections in RTRs [21]. Among recipients of hematopoietic cell transplants and their unrelated donors, the *TLR4* 896 A>G and *TLR4* 1196 C>T SNPs were reported to be possibly associated with the risk factors of invasive aspergillosis that included HCMV seropositivity [25]. No previous study showed any involvement of *TLR9* 2848 G>A SNP in HCMV infection, although the polymorphism was reported to be associated with other pregnancy disorders, including the mother-to-child transmission (MTCT) of human immunodeficiency virus type 1 (HIV-1), toxoplasmic retinochoroiditis, as well as cervical cancer [26–30]. So far, it is unclear whether distinct SNPs, located within *TLR* genes, could be expected as correlated with HCMV infections in different populations.

In the reported study, we developed multiplex nested PCR-RFLP to define the genetic status at *TLR4* 896 A>G, 1196 C>T and *TLR9* 2848 G>A SNPs among fetuses and neonates. The incidence rates of genotypes and alleles at three *TLR* SNPs were determined in the groups of fetuses and newborns, both with congenital cytomegaly and in non-infected controls.

## Materials and Methods

The study was performed retrospectively on samples, obtained from 18 fetuses and newborns, infected congenitally with HCMV, and for 19 control cases without infection that were collected at the Department of Fetal-Maternal Medicine and Gynecology of the Polish Mother’s Memorial Hospital—Research Institute in Lodz between the years 2000 and 2012. Asymptomatic cytomegaly was observed in nine offsprings, while symptomatic disease was identified in other nine patients.

Clinical materials were selected to molecular analyses, depending on their availability and included amniotic and/or ascitic (two samples) fluids, umbilical cord blood and amniotic membranes, as well as whole blood, plasma and samples of urine of newborns. The amniotic fluid samples were obtained during amniocentesis in pregnant women, treated at the Institute. Fetal umbilical cord blood samples, membranes, as well as neonatal blood and urine specimens, were collected promptly after birth. Fetuses and newborns were preliminary indicated as possibly infected with HCMV, based on the ultrasound markers, related to cytomegaly, as well as to serological features of the recent infection in pregnant women and HCMV DNAemia in their blood and urine samples. Congenital diseases were confirmed by the presence of viral DNA, assayed in body fluids from the fetuses and newborns. The study was approved by the Research Ethics Committee at the Polish Mother’s Memorial Hospital—Research Institute. For the molecular analyses, we used the samples, collected previously for diagnostic purposes and anonymized in the study. An informed consent forms were signed by pregnant women participating in the study.

### Serological tests

Blood specimens were obtained from pregnant women, participating in the study, collected by venipuncture during the first visit to the Institute. Serum samples were obtained by centrifugation and then stored at 4°C before analysis. Serological tests were performed at the Department of Clinical Microbiology at the Institute.

Serological screening was based on Eti-Cytok G-Plus and Eti-Cytok M-Reverse Plus tests (Diasorin/Biomedica, Italy), used between the years 2000 and 2001, VIDAS CMV IgG and IgM tests (bioMérieux, France)—used between 2001 and 2006, anti-CMV IgG and IgM tests (Diasorin/Biomedica, Italy)—used between 2006 and 2011 years and ELFA assays—from the year 2012. The pregnant women were infected with HCMV in case of IgG seroconversion during pregnancy, in the presence of IgG and IgM specific antibodies or a low IgG avidity index. In those patients and their offsprings, viral DNA was assayed, using a real-time Q PCR for viral *UL55* gene in blood, urine and amniotic fluids.

### DNA isolation

For genetic studies of fetuses and neonates, genomic and/or viral DNA was extracted from fetal amniotic and/or ascitic fluid, membrane and umbilical cord blood samples as well as neonatal whole blood, plasma and urine specimens, using a QIAamp DNA Mini Kit (QIAGEN, Hilden, Germany), according to the manufacturer’s guidelines. Extracted DNA was diluted in 100 μl of elution buffer and stored at -20°C until molecular analyses.

### Detection and quantification of HCMV DNA

The presence and amount of HCMV DNA in the study specimens were estimated on the basis of the real-time Q PCR assay for detection of viral *UL55* gene fragment, as described previously [31, 32]. The standard curves were obtained from serial 10-fold dilutions from 10^5^ to 1 plasmid DNA, containing the entire HCMV *UL55* open reading frame [33]. The amplification was performed by a 7900 HT Fast Real-Time PCR System (Applied Biosystems, USA).

### Determination of SNPs located in *TLR4* and *TLR9* genes

**Fig 1:**
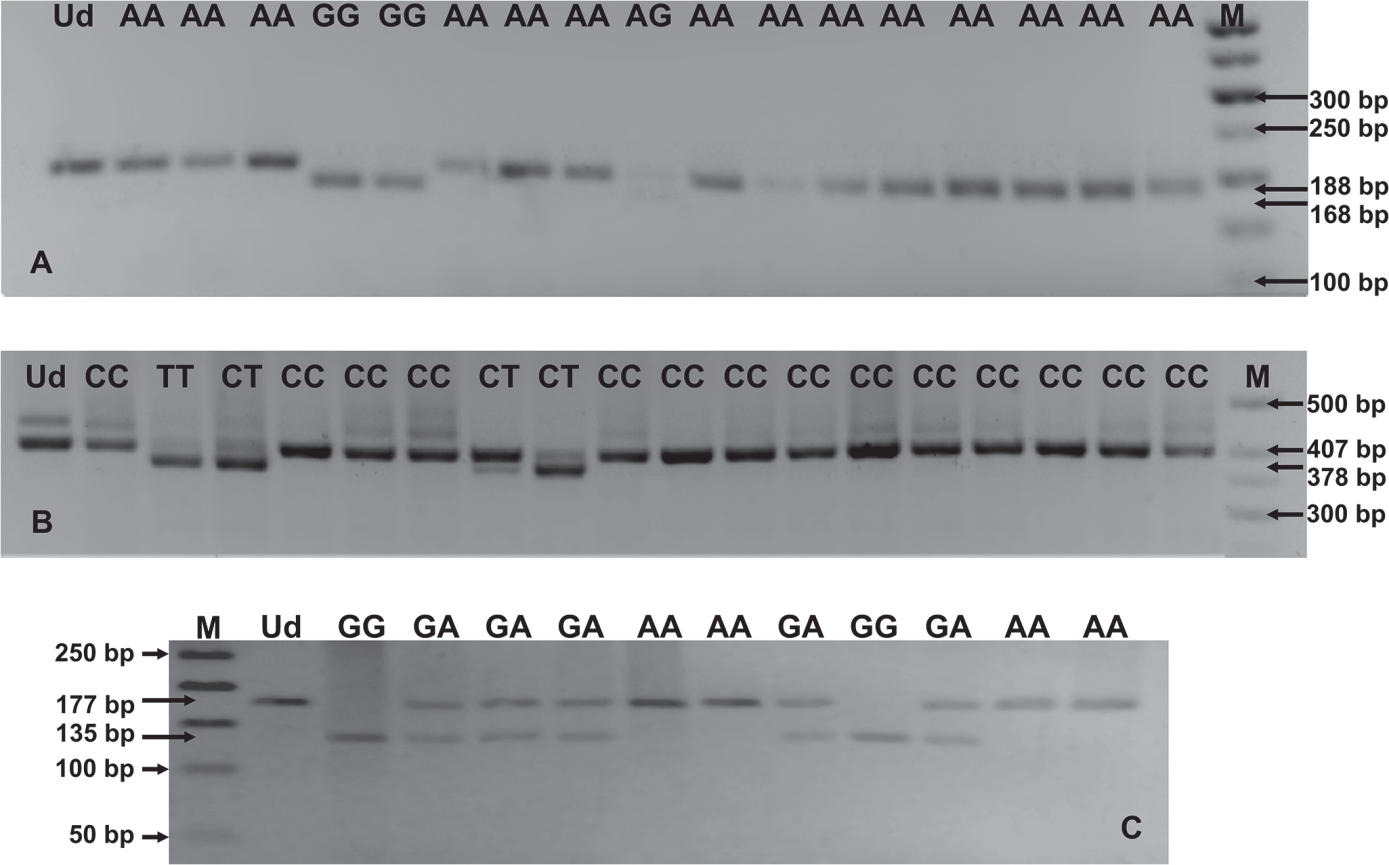
PCR-RFLP analysis of genotypes at *TLR4* (A, B) and *TLR9* (C) SNPs. RFLP products were resolved in 2% agarose gels, stained with ethidium bromide; A. *TLR4* 896 A>G SNP; B. *TLR4* 1196 C>T SNP; C. *TLR9* 2848 G>A SNP. The numbers on the side of electropherograms indicate the lengths of separated DNA fragments. M—50 bp DNA marker; Ud—undigested PCR product; AA, AG, GG, GA, CC, CT or TT—genotypes at analyzed *TLR* SNPs.

We developed a multiplex nested PCR assay to discriminate *TLR4* 896 A>G and 1196 C>T SNPs and *TLR9* 2848 G>A SNP. GenBank accession numbers for gene coding sequences, external and internal primer sequences, amplicon lengths and the annealing temperatures, used to amplify internal fragments of particular genes are presented in Table 1. External primers were designed using Vector NTI Suite 5.5 software, whereas internal primers were adapted from published articles [34–36]. Multiplex nested PCR assays were performed using HotStarTaq Master Mix Kit (QIAGEN, Hilden, Germany). In multiplex PCR assays, all the 6 external primers for the three analyzed SNPs were put into one PCR tube. Multiplex PCR conditions were as follows: an initial activation for 15 min at 95°C and for 40 cycles of repeated denaturation at 94°C for 30 sec, annealing at 52°C for 1 min and extension at 72°C for 2 min, and final extension at 72°C for 10 min. Similar reaction parameters, but with distinct annealing temperatures appropriate for particular internal primer pairs, were used for nested PCR assays (see Table 1). Amplicons from multiplex nested PCRs were resolved by electrophoresis on 1% agarose gels and then digested with appropriate endonucleases. In order to determine *TLR4* 896 A>G SNP, we used NcoI enzyme, *TLR4* 1196 C>T SNP—HinfI and to *TLR9* 2848 G>A SNP—BstUI. Digestions were performed in tubes distinct for different SNPs, using reaction mixtures containing 10 μl of the PCR product for particular gene fragment, 10 U of appropriate endonuclease, 1 x concentrated buffers for endonuclease and distilled nuclease-free water, added to final reaction volume of 20 μl. The PCR products were digested overnight at 37°C with NcoI or HinfI endonucleases and at 60°C with BstUI. The digestion products were resolved on 2% agarose gels. *TLR* SNPs and genotypes were discriminated by length of restriction fragments, just as it was described in the previous papers ([34–36]; see Table 2, Fig 1). Genotypes at *TLR4* 896 A>G and 1196 C>T SNPs were assessed for 33, out of the 37 studied fetuses and neonates, while the genetic variants at *TLR9* 2848 G>A SNP were assessed for all 37 analyzed offsprings.

**Table 1:** Primer sequences, annealing temperatures and amplicon lengths, obtained in multiplex nested PCR assay for SNPs in the *TLR4* and *TLR9* genes.

Gene	GenBank Accession No.^a^	SNP^b^ name	Primer sequences (5'-3')		Annealing temperature [°C]	Amplicon length (bps)^c^
***TLR4***	NG_011475	896 A>G	External	For: AAAACTTGTATTCAAGGTCTGGC	52	355
		(rs4986790)		Rev: TGTTGGAAGTGAAAGTAAGCCT		
			Internal	For: AGCATACTTAGACTACTACCTCCATG	61	188
				Rev: AGAAGATTTGAGTTTCAATGTGGG		
		1196 C>T	External	For: AGTTGATCTACCAAGCCTTGAGT	52	510
		(rs4986791)		Rev: GGAAACGTATCCAATGAAAAGA		
			Internal	For: GGTTGCTGTTCTCAAAGTGATTTTGGGAGAA	59	407
** **				Rev: ACCTGAAGACTGGAGAGTGAGTTAAATGCT		
***TLR9***	EU170539	2848 G>A	External	For: GTCAATGGCTCCCAGTTCC	52	292
		(rs352140)		Rev: CATTGCCGCTGAAGTCCA		
			Internal	For: AAGCTGGACCTCTACCACGA	59	177
				Rev: TTGGCTGTGGATGTTGTT		

^a^ No., number.

^b^ SNP, single nucleotide polymorphism.

^c^ bps, base pairs.

**Table 2:** Length of restriction fragments and genotypic profiles.

***TLR* SNP** ^a^	**Restriction enzyme**	**Profile (bps)** ^b^
***TLR4* 896 A>G**	NcoI	AA: 188
		AG: 188, 168, 20
		GG: 168, 20
***TLR4* 1196 C>T**	HinfI	CC: 407
		CT: 407, 378, 29
		TT: 378, 29
***TLR9* 2848 G>A**	BstUI	GG: 135, 42
		GA: 177, 135, 42
		AA: 177

^a^ SNP, single nucleotide polymorphism.

^b^ bps, base pairs.

**Fig 2:**
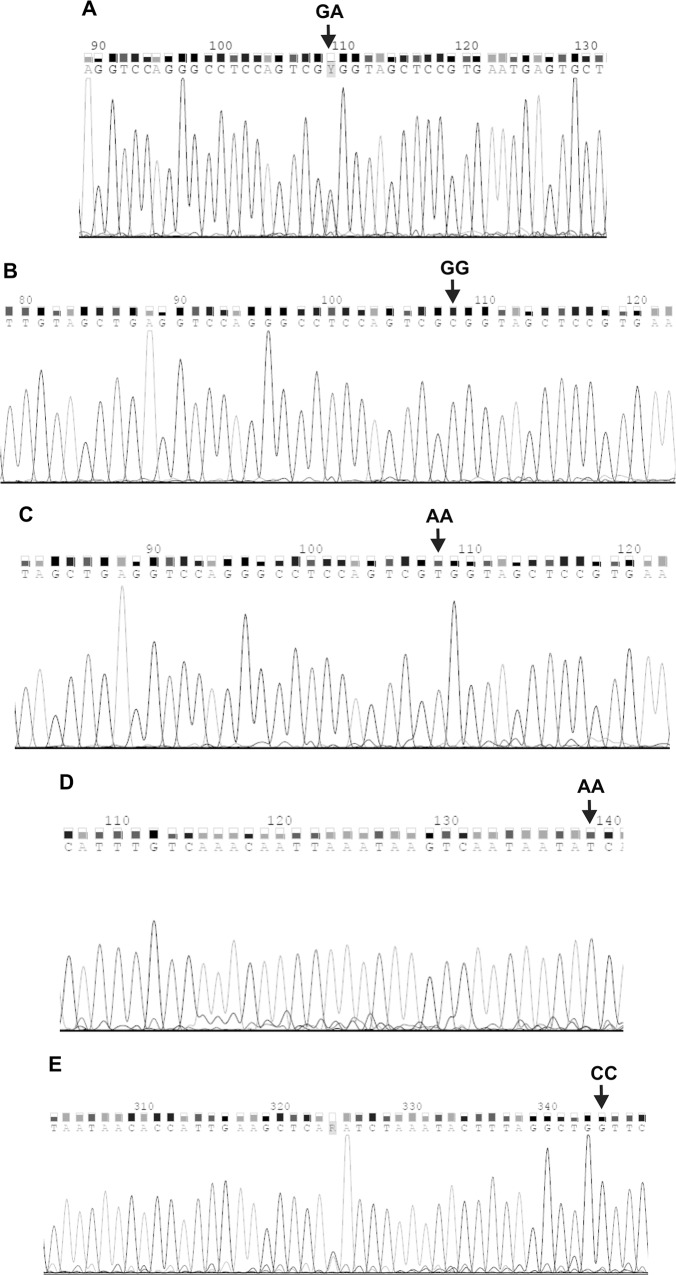
DNA sequences, comprising polymorphic sites at *TLR4* (A, B) and *TLR9* (C-E) SNPs. Reverse strand sequences were determined for all the analyzed amplicons of *TLR* gene fragments; A. *TLR4* 896 A>G SNP; B. *TLR4* 1196 C>T SNP; C-E. *TLR9* 2848 G>A SNP. AA, CC, GG or GA—genotypes at described *TLR* SNPs.

The randomly selected samples, representative for *TLR* genotypes, were then verified by sequencing of PCR products with the Sanger method. The sequencing was performed for three AA homozygotes at *TLR* 896 A>G SNP, two CC homozygotes at *TLR* 1196 C>T SNP, as well as for two GG homozygotes, five GA heterozygotes and two AA homozygotes at *TLR9* 2848 G>A SNP. The chromatograms, illustrating DNA sequences for different *TLR* SNPs, are shown in Fig 2. A sequence analysis was performed using the BLASTN program, enabling the alignment of two (or more) sequences. A sequencing chromatogram analysis was performed using the Sequence Scanner 1.0 and the Chromas Lite 2.1.1 softwares.

### Statistical analysis

The frequencies of genotypes and alleles at *TLR* SNPs, observed among HCMV infected and control fetuses and newborns, were assessed by means of descriptive statistics. The study groups were analyzed for Hardy-Weinberg (H-W) equilibrium, linkage disequilibrium (LD) and haplotypes, using the SNPStats software (http://bioinfo.iconcologia.net/en/SNPStats_web). The differences in genotype and allele distribution, observed between cases and controls, as well as between symptomatic and asymptomatic cases, were determined by cross-tabulation, Pearson's Chi-squared or Fisher's exact tests, as well as the logistic regression model. The analysis of haplotypes at *TLR4* SNPs, as well as multiple-SNP analysis, was performed using the Expectation Maximization (EM) algorithm. All the results were defined as statistically significant at *P*≤0.050. A part of the statistical analysis was also performed using the NCSS 97 software.

## Results

### Hardy-Weinberg equilibrium, linkage disequilibrium

In the fetuses and newborns, infected with HCMV, the frequencies of genotypes at all the analyzed SNPs were in H-W equilibrium (*P* = 1.000 for genotypes at *TLR4* SNPs and *P* = 0.055 for *TLR9* SNP). In the control group, the frequencies of genotypes at *TLR4* 1196 C>T and *TLR9* 2848 G>A SNPs were in H-W equilibrium (*P* = 0.270 and *P* = 0.640, respectively), while *TLR4* 896 A>G SNP was not (*P*≤0.050). In case of *TLR4* 896 A>G SNP, the observed frequency of heterozygotes was lower, as compared to the values expected from the H-W equilibrium (4.31% *vs*. 23.92%, respectively). For the other SNPs, analyzed in the study, the observed and expected frequencies of distinct genotypes were similar. *TLR4* 896 A>G and 1196 C>T SNPs were in linkage disequilibrium, both in the infected and control groups (*P*≤0.050).

### Genotypes at *TLR4* 896 A>G, 1196 C>T and *TLR9* 2848 G>A SNPs

In the infected fetuses and newborns, the frequencies of the AA and AG genotypes at *TLR4* 896 A>G SNP were 93.3% (14/15) and 6.7% (1/15), respectively (see Table 3). In case of *TLR4* 1196 C>T SNP, only the CC genotype was observed (100%; 15/15). For *TLR9* 2848 G>A SNP, the genotypes GG, GA and AA occurred in 11.1% (2/18), 77.8% (14/18) and 11.1% (1/18) of fetuses and newborns, respectively. In the control group, the frequencies of AA, AG and GG genotypes at *TLR4* 896 A>G were 83.3% (15/18), 5.6% (1/18) and 11.1% (2/18), respectively. The CC, CT and TT genotypes at *TLR4* 1196 C>T occurred in 77.8% (14/18), 16.7% (3/18) and 5.6% (1/18) of cases, respectively. For *TLR9* SNP, the frequencies of GG, GA and AA genotypes were 21.1% (4/19), 42.1% (8/19) and 36.8% (7/18), respectively. The distribution of genotypes at *TLR4* 1196 C>T and *TLR9* G>A SNPs was significantly different between the infected and the control fetuses and neonates, while in case of *TLR4* 896 A>G, no such difference was observed. The frequency of the CC genotype at *TLR4* 1196 C>T was significantly higher in the infected fetuses and neonates than in the control cases (OR 1.00; *P*≤0.050, both in the dominant and overdominant models). Considering *TLR9* SNP, the GA heterozygotes, when compared to AA and GG homozygotes, were significantly more frequent in the infected patients than in the controls, and increased the risk of HCMV infection (OR 4.81, 95% CI 1.14–20.25; *P*≤0.050 in the overdominant model). The comparison of the distribution of simultaneous carriers of the CC and GA genotypes at *TLR4* 1196 and *TLR9* SNPs, respectively, between the infected and the control groups, showed a significantly higher frequency of the analyzed variants among fetuses and neonates with congenital cytomegaly (80.0% *vs*. 27.8%; *P*≤0.050; Fisher’s exact test). Taking into account the outcome of congenital cytomegaly, no difference was observed in the distribution of the analyzed genotypes at all the three *TLR* SNPs between symptomatic and asymptomatic cases (see S1 Table).

**Table 3:** Single-SNP analysis of the relationship between *TLR* polymorphisms and congenital HCMV infection.

Gene polymorphism	Genetic model	Genotype	Genotype frequencies; n (%)^a^		OR^b^ (95% CI)^c^	*P*-value^d^
			Infected cases	Controls		
***TLR4* 896 A>G**	Codominant	AA	14 (93.3%)	15 (83.3%)	1.00	0.280
		AG	1 (6.7%)	1 (5.6%)	1.07 (0.06–18.82)	
		GG	0 (0%)	2 (11.1%)	0.00 (0.00-NA)^e^	
	Dominant	AA	14 (93.3%)	15 (83.3%)	1.00	0.370
		AG-GG	1 (6.7%)	3 (16.7%)	0.36 (0.03–3.85)	
	Recessive	AA-AG	15 (100%)	16 (88.9%)	1.00	0.110
		GG	0 (0%)	2 (11.1%)	0.00 (0.00-NA)	
	Overdominant	AA-GG	14 (93.3%)	17 (94.4%)	1.00	0.890
		AG	1 (6.7%)	1 (5.6%)	1.21 (0.07–21.22)	
***TLR4* 1196 C>T**	Codominant	CC	15 (100%)	14 (77.8%)	1.00	0.070
		CT	0 (0%)	3 (16.7%)	0.00 (0.00-NA)	
		TT	0 (0%)	1 (5.6%)	0.00 (0.00-NA)	
	Dominant	CC	15 (100%)	14 (77.8%)	1.00	≤ 0.050
		CT-TT	0 (0%)	4 (22.2%)	0.00 (0.00-NA)	
	Recessive	CC-CT	15 (100%)	17 (94.4%)	1.00	0.270
		TT	0 (0%)	1 (5.6%)	0.00 (0.00-NA)	
	Overdominant	CC-TT	15 (100%)	15 (83.3%)	1.00	≤ 0.050
		CT	0 (0%)	3 (16.7%)	0.00 (0.00-NA)	
***TLR9* 2848 G>A**	Codominant	AA	2 (11.1%)	7 (36.8%)	1.00	0.072
		GA	14 (77.8%)	8 (42.1%)	6.12 (1.02–36.89)	
		GG	2 (11.1%)	4 (21.1%)	1.75 (0.17–17.69)	
	Dominant	AA	2 (11.1%)	7 (36.8%)	1.00	0.062
		GA-GG	16 (88.9%)	12 (63.2%)	4.67 (0.82–26.60)	
	Recessive	AA-GA	16 (88.9%)	15 (79.0%)	1.00	0.410
		GG	2 (11.1%)	4 (21.1%)	0.47 (0.07–2.94)	
	Overdominant	AA-GG	4 (22.2%)	11 (57.9%)	1.00	≤ 0.050
		GA	14 (77.8%)	8 (42.1%)	4.81 (1.14–20.25)	

^a^ n, number of tested fetuses and newborns;

^b^ OR, odds ratio;

^c^ 95% CI, confidence interval;

^d^ logistic regression model; *P*≤0.050 is considered as significant;

^e^ NA, not analyzed.

### Frequencies of alleles residing within *TLR4* and *TLR9* polymorphic sites

In the fetuses and newborns with HCMV infection, considering *TLR4* 896 A>G polymorphic site, the frequency of allele A was 96.7% (29/30), while of G—3.3% (1/30; see Table 4). At the region of *TLR4* 1196 C>T SNP, only the allele C was observed (100%; 30/30). In case of *TLR9* SNP, both G and A allelles occurred with the frequency of 50.0% (18/36). Among the control cases, in *TLR4* 896 A>G site, the frequencies of the A and G alleles were 86.1% (31/36) and 13.9% (5/36), respectively. For the *TLR4* 1196 C>T region, the C and T allelles occurred with the frequencies of 86.1% (31/36) and 13.9% (1/36), respectively. In case of *TLR9* SNP, the frequencies of the G and A alleles were 42.1% (16/38) and 57.9% (22/38), respectively. Significant differences were determined in the frequencies of alleles at *TLR4* 1196 C>T polymorphic site between the congenitally infected and non-infected fetuses and newborns (χ^2^ = 4.8; *P*≤0.050). At the other analyzed polymorphic sites, the allele incidence rates were similar, both in the case group and among the controls.

**Table 4:** Distribution of the alleles, located at *TLR4* and *TLR9* polymorphic sites.

Gene polymorphism and alleles		No.^a^ of carriers with *TLR* alleles (%)		*P*-value^b^
		Cases	Controls	
***TLR4* 896 A>G**				
	A	29 (96.7)	31 (86.1)	0.137
	G	1 (3.3)	5 (13.9)	
***TLR4* 1196 C>T**				
	C	30 (100)	31 (86.1)	≤ 0.050
	T	0 (0)	5 (13.9)	
***TLR9* 2848 G>A**				
	G	18 (50.0%)	16 (42.1%)	0.496
	A	18 (50.0%)	22 (57.9%)	

^a^ No.—number;

^b^ Pearson's Chi-squared test; *P*≤0.050 is considered significant.

### Multiple-SNP analysis of *TLR* polymorphisms in fetuses and neonates

In the infected and control fetuses and neonates, the most common haplotype for *TLR4* 896 A>G and 1196 C>T SNPs was AC (96.2% and 83.3% for the infected and control cases, respectively). The GC haplotype was observed at low frequencies in both study groups (3.9% and 2.8%, respectively), while GT and AT haplotypes were found only in the control group (with the frequencies of 11.1% and 2.8%, respectively). The GC haplotype was significantly associated with the occurrence of HCMV infection and the increased risk of congenital cytomegaly (OR 8.2 x 10^8^; *P*≤0.0001). No relationship was observed between haplotypes at *TLR4* SNPs and congenital HCMV infection. Taking into account the alleles, present at all three analyzed SNPs, a simultaneous occurrence of A, C and G variants at *TLR4* 896 A>G, 1196 C>T and *TLR9* SNPs was most frequently observed in the infected fetuses and newborns (57.7% *vs*. 37.8% in the infected and control groups, respectively), while A, C and A variants at the relevant SNPs were estimated as the most frequent in the control group (45.5% *vs*. 38.5% in control and infected groups, respectively). In addition, GCA variants at the three analyzed *TLR* SNPs were significantly associated with congenital HCMV infection (3.9% *vs*. 2.8% in the infected and control fetuses and neonates, respectively; OR 6.5 x 10^12^; *P*≤0.0001). The multiple GTA, GTG and ATG variants were observed only in the control group (7.2%, 3.8% and 2.8%, respectively). No relationship was observed between all the other multiple genetic variants and the HCMV infection. Considering the outcome of congenital cytomegaly, the ACA variants were more frequent among symptomatic than asymptomatic cases.

## Discussion

In the reported study, we determined that GA heterozygotic status at *TLR9* 2848 SNP was associated with susceptibility of fetuses and newborns to congenital infection with HCMV and an increased—by 4.81 times—risk of the infection. So far, only one study, performed in Japanese children, congenitally infected with HCMV, provide a report, specifying the prevalence rates of genotypes and alleles at this polymorphic site [22]. Similarly to our results, the frequencies of GA genotypes at *TLR9* 2848 SNP were higher in the congenitally infected children than in the control cases [22]. However, the difference in the prevalence of the analyzed genotypes at *TLR9* SNP was not significant and none of the genotypic variants were shown as a marker, predisposing to congenital infection or HCMV disease [22]. Another study, performed to analyze the involvement of *TLR9* 2848 SNP in susceptibility to meningococcal meningitis, also showed similar frequencies of GG, GA and AA genotypes, with the highest occurrence of heterozygotes [37]. Despite the rather small number of studies on the relationship between *TLR9* 2848 SNP and congenital infection with HCMV, a number of reports inform that the contribution of *TLR9* to the occurrence and development of HCMV infections is available [13, 16]. Plasmacytoid dendritic cells (DCs), infected with HCMV, showed partial maturation, as well as elevated expression of MHC class II, cluster of differentiation 83 (CD83) and TLR9 [16]. The treatment of pDCs with ligands for TLR (CpG) resulted in an inhibition of cytokine expression, suggesting some contribution of TLR7 or TLR9 in their regulation [16]. Considering the activity of TLR9 in the development of HCMV infection, the involvement of GA heterozygotic status at *TLR9* 2848 SNP seems to be possible in an altered response against the virus, including the changed expression of proinflammatory cytokines. Since *TLR9* 2848 SNP is not associated with either an amino acid change or the alteration of the regulatory site, other modifications, linked to the described mutation, might be involved in the associated lesions [37]. In our study, we showed that the simultaneous occurrence of the G, C and A alleles at the analyzed *TLR4* 896 A>G, 1196 C>T and *TLR9* 2848 SNPs, respectively, was significantly associated with HCMV congenital infection. The ACA variants at described SNPs were significantly more frequent among the fetuses and neonates with symptomatic, rather than asymptomatic cytomegaly. So far, no other study has reported similar analyses of the influence of multiple *TLR4* and *TLR9* SNPs on the congenital HCMV infection, although it seems to be an important trend in the search for the related molecular markers.

Taking into consideration *TLR4* 1196 C>T SNP, we showed the CC genotype at the *locus* as significantly associated with HCMV infection in fetuses and neonates. Additionally, the C allele at 1196 SNP was significantly more frequent among the infected fetuses and neonates *vs*. the control ones. Therefore, we suggest that the C allele might predispose to the acquisition and development of congenital infection with HCMV. Another study, designed and attempted for adult RTRs, showed a rather marginal association between the mutations, either at *TLR4* 896 A>G or 1196 C>T SNPs and HCMV disease [23]. Similarly to our control fetuses and neonates, the frequencies of mutant heterozygotes and homozygotes were low, both at *TLR4* SNPs in RTRs [23]. In contrast to our results, the polymorphic status at *TLR4* SNPs was significantly more frequent in patients with HCMV disease than in those without. Such discrepancies might have been caused by age differences between the two distinct study groups. In fetuses and neonates, immunity, including TLRs expression is still immature [38–41]. In neonates, when compared to adults, a lower level of functional TLR4 on monocytes resulted in a relatively low expression of TNF-α after LPS stimulation [41]. During the first year of neonatal life, altered expression levels were shown, both for TLR4 and TLR9 molecules, as well as proinflammatory cytokines, induced by TLR signaling [40]. The outcomes, as reported in this paper, may suggest that, in the fetuses and neonates, the wild type C allele at *TLR4* 1196 C>T SNP, in combination with the alterations related to immature immune response, may predispose to the development of congenital infection with HCMV. Additionally, other lesions, located within *TLR4* gene, also may have been related to the predisposition to HCMV infections. So far, some molecular mechanisms have been reported, underlying the contribution of *TLR4* 896 A>G and 1196 C>T SNPs in the altered immune response [42–44]. Both *TLR4* SNPs were shown to impair TLR4/MD2 dimerization necessary to activate downstream signaling [43]. The TLR4/MD2/CD14 complex has been reported to be involved in HCMV-induced signaling pathways [44]. In other studies, a mutant variant at 896 A>G SNP was suggested to affect TLR4 dimerization without changing its expression [42]. The previous study of human embryonic kidney cells, transfected with wild type or mutant *TLR4* variants showed failed LPS-induced signaling in carriers of the 896 A>G SNP but not of the 1196 C>T mutation [42]. *TLR4* 896 G>A and 1196 C>T polymorphisms were reported to cause co-segregating Asp299Gly and Thr399Ile missense mutations, respectively, that are located in the ectocellular domain of the encoded protein [45, 46]. Both mutations were observed to diminish the TLR4 response to its ligand LPS in humans, while the Asp299Gly alteration was shown to interrupt the response [45]. In addition, that mutation resulted in a disruption of TLR4 α-helical protein structure and in extension of β-strand [47]. In our study, the analyzed *TLR4* SNPs were in linkage disequilibrium and, in addition, the GC haplotype, at the described *loci*, was correlated with HCMV infection and occurred significantly more frequently among symptomatic cytomegaly cases rather than among the asymptomatic ones. This may suggest that the presence of both, the G allele at 896 A>G *locus* and the C allele at 1196 C>T *locus*, may have been a significant cause of impaired TLR4 functions in fetuses and neonates. Taking into account the previous data, it seems possible that the occurrence of the minor allele at the *TLR4* 896 A>G polymorphic site, resulting in conformational changes of TLR4 molecule, may have been the main cause of disrupted immune response after infection with HCMV in the analysed fetuses and newborns. We suggest that a confirmation of our findings in a larger study, as well as in an animal model, would be valuable, appropriate and desirable.

Our outcomes demonstrate that *TLR4* 896 A>G and 1196 C>T, as well as *TLR9* 2848 G>A polymorphisms might contribute to the development of congenital infection with HCMV in fetuses and neonates. At *TLR9* 2848 SNP, the GA heterozygotic status was associated with the HCMV infection in the overdominant model and increased the risk of congenital cytomegaly by 4.81 times. Additionally, the GCA variants at *TLR4* 896 A>G, 1196 C>T and *TLR9* 2848 G>A SNPs were significantly associated with HCMV infection. The observed linkage disequilibrium for the analyzed SNPs, located in *TLR4* gene, as well as the significant association of the GC haplotype at those SNPs with the infection, suggested that the simultaneous presence of the G allele at 896 SNP and the C allele at 1196 SNP may have affected the function of *TLR4* in the immunity against HCMV in the fetuses and neonates. Particularly, the mutant allele at *TLR4* 896 A>G SNP seems to be possibly involved in the disruption of TLR4 activity; however, it should be investigated and verified in further mechanistic studies.

## Supporting Information

S1 Table Single-SNP analysis of the relationship between *TLR* polymorphisms and cytomegaly outcome. ^a^ n, number of tested fetuses and newborns; ^b^ OR, odds ratio; ^c^ 95% CI, confidence interval; ^d^ logistic regression model; *P*≤0.050 is considered as significant; ^e^ NA, not analyzed
(DOC)
