# Supplementary material for: TLR9 2848 GA Heterozygotic Status Possibly Predisposes Fetuses and Newborns to Congenital Infection with Human Cytomegalovirus
Source: PLoS One. 2015 Apr 6;10(4):e0122831. doi: 10.1371/journal.pone.0122831 (PMC4386761; doi:10.1371/journal.pone.0122831)
Supplement: S1 Table — a n, number of tested fetuses and newborns; b OR, odds ratio; c 95% CI, confidence interval; d logistic regression model; P≤0.050 is considered as significant; e NA, not analyzed (DOC) [file pone.0122831.s001.doc]

| **Supporting Information**  **Table S1.** Single-SNP analysis of the relationship between *TLR* polymorphisms and cytomegaly outcome | | | | | | |
| --- | --- | --- | --- | --- | --- | --- |
|  |  |  |  |  |  |  |
| **Gene polymorphism** | **Genetic model** | **Genotype** | **Genotype frequencies among infected cases; n (%)**^a^ | | **OR**^b^ **(95% CI)**^c^ | ***P*-value**^d^ |
|  |  |  | **Symptomatic** | **Asymptomatic** |  |  |
|  |  |  |  |  |  |  |
| ***TLR4* 896 A>G** | - | AA | 7 (87.5%) | 7 (100%) | 1.00 | 0.250 |
|  |  | AG | 1 (12.5%) | 0 (0%) | NA^e^ (0.00-NA) |  |
|  |  |  |  |  |  |  |
| ***TLR9* 2848 G>A** | Codominant | GG | 0 (0%) | 2 (22.2%) | 1.00 | 0.220 |
|  |  | GA | 8 (88.9%) | 6 (66.7%) | 1.33 (0.07-25.91) |  |
|  |  | AA | 1 (11.1%) | 1 (11.1%) | 0.00 (0.00-NA) |  |
|  | Dominant | AA | 1 (11.1%) | 1 (11.1%) | 1.00 | 1.000 |
|  |  | GA-GG | 8 (88.9%) | 8 (88.9%) | 1.00 (0.05-18.92) |  |
|  | Recessive | AA-GA | 9 (100%) | 7 (77.8%) | 1.00 | 0.082 |
|  |  | GG | 0 (0%) | 2 (22.2%) | 0.00 (0.00-NA) |  |
|  | Overdominant | GG-AA | 1 (11.1%) | 3 (33.3%) | 1.00 | 0.250 |
|  |  | GA | 8 (88.9%) | 6 (66.7%) | 4.00 (0.33-48.66) |  |

^a^ n, number of tested fetuses and newborns; ^b^ OR, odds ratio; ^c^ 95% CI, confidence interval;
^d^ logistic regression model; *P*≤0.050 is considered as significant; ^e^ NA, not analyzed
